# First record of *Rickettsia bellii* in *Amblyomma varium* and *Amblyomma goeldii* from the Eastern Amazon

**DOI:** 10.1590/S1984-29612025043

**Published:** 2025-08-13

**Authors:** Izabela Mesquita Araújo, Kelly Karoline Gomes do Nascimento, Mateus Borges Silva, Isis Abel, Ana Cristina Mendes-Oliveira, Bruna de Azevedo Baêta, Claudia Bezerra da Silva, Matheus Dias Cordeiro

**Affiliations:** 1 Universidade Federal Rural do Rio de Janeiro - UFRRJ, Instituto de Veterinária - IV, Departamento de Parasitologia – DPA, Seropédica, Rio de Janeiro, Brasil; 2 Universidade Federal do Pará – UFPA, Faculdade de Medicina Veterinária, Castanhal, Pará, Brasil; 3 UFPA, Instituto de Ciências Biológicas - ICB, Belém, PA, Brasil; 4 Universidade Federal Rural do Rio de Janeiro - UFRRJ, Instituto de Veterinária - IV, Departamento de Epidemiologia e Saúde Pública – DESP, Seropédica, Rio de Janeiro, Brasil

**Keywords:** Etiological agents, ticks, Amazon rainforest, Brazil, Agentes etiológicos, carrapatos, Floresta Amazônica, Brasil

## Abstract

This study investigated the presence of *Rickettsia* spp., *Ehrlichia* spp., *Anaplasma* spp., and *Borrelia* spp. DNA in questing ticks collected from a forest fragment under constant anthropogenic pressure in the state of Pará, Brazil. The fragment was divided into three zones: interior, edge, and surrounding matrix. Ticks were collected in all zones using flannel drag sampling, flannel leggings, and CO_2_-baited traps. Nymphs and adults were morphologically identified using taxonomic keys, while larvae were subjected to molecular identification. DNA extracted from the ticks was tested for the target pathogens using polymerase chain reaction (PCR). In total, 561 questing ticks (525 larvae, 29 nymphs, and 7 adults) were collected, representing eight species: *Amblyomma cajennense sensu stricto*, *Amblyomma calcaratum*, *Amblyomma geayi*, *Amblyomma goeldii*, *Amblyomma humerale*, *Amblyomma naponense*, *Amblyomma pacae*, and *Amblyomma varium*. *Rickettsia bellii* was detected in one *A. varium* larva and one *A. goeldii* larva. All samples tested negative for *Ehrlichia* spp., *Anaplasma* spp., and *Borrelia* spp. This is the first report of *R. bellii* in *A. varium* and *A. goeldii* in Brazil.

## Resumo

Este estudo investigou a presença de DNA de *Rickettsia* spp., *Ehrlichia* spp., *Anaplasma* spp. e *Borrelia* spp. em carrapatos em fase de vida livre coletados de um fragmento florestal sob constante pressão antrópica no estado do Pará, Brasil. O fragmento foi dividido em três áreas: interior, borda e matriz circundante. Os carrapatos foram coletados em todas as áreas usando um arrasto de flanela, perneiras de flanela e armadilhas com isca de CO_2_. Ninfas e adultos foram identificados morfologicamente usando chaves taxonômicas, enquanto larvas foram submetidas à identificação molecular. DNA extraído dos carrapatos foi testado para os patógenos-alvo usando reação em cadeia pela polimerase (PCR). No total, foram coletados 561 carrapatos (525 larvas, 29 ninfas e 7 adultos), representando oito espécies: *Amblyomma cajennense sensu stricto*, *Amblyomma calcaratum*, *Amblyomma geayi*, *Amblyomma goeldii*, *Amblyomma humerale*, *Amblyomma naponense*, *Amblyomma pacae* e *Amblyomma varium*. *Rickettsia bellii* foi detectada em uma larva de *A. varium* e uma larva de *A. goeldii*. Todas as amostras apresentaram resultados negativos para *Ehrlichia* spp., *Anaplasma* spp. e *Borrelia* spp. Este é o primeiro relato de *R. bellii* em *A. varium* e *A. goeldii* no Brasil.

## Introduction

The Amazon Rainforest is the world's most biodiverse tropical forest (Ellwanger et al., 2020). However, anthropogenic activities, particularly deforestation, continue to threaten the biodiversity. Forest fragmentation creates distinct forest patches surrounded by human-altered environments such as agricultural lands, pastures, and settlements (Forman, 1995). The edges of these forest fragments are ecologically significant, revealing imbalances caused by human intervention that serve as interfaces between wildlife and human populations. Studies have shown that human activities in forested areas increase direct contact between humans and wild species, including tick vectors, thereby promoting the emergence or re-emergence of zoonotic diseases (Ellwanger et al., 2020).

Ticks, as hematophagous ectoparasites, pose significant public health concerns because of their ability to transmit various bacterial, protozoan, and viral pathogens, often harboring multiple agents simultaneously (Madison-Antenucci et al., 2020).

In Brazil, tick fauna currently comprises 78 species, predominantly belonging to the genus *Amblyomma* (Dantas-Torres et al., 2019). Studies of tick fauna in the northern region of Brazil have mainly focused on the Western Amazon, particularly in Rondônia (Labruna et al., 2005; Martins et al., 2010, 2016). However, studies on tick fauna and their transmitted pathogens in the Eastern Amazon remain limited, particularly in the state of Pará (Martins et al., 2014). This study aimed to conduct molecular investigations of *Rickettsia* spp., *Ehrlichia* spp., *Anaplasma* spp., and *Borrelia* spp. in questing ticks collected from a forest fragment frequently accessed by a human settlement in the state of Pará, Brazil.

## Material and Methods

The questing ticks were captured in a forest fragment in Vila Ananim (01°06’29.1”S and 047°19’52.9”W), located in the municipality of Peixe-Boi, Pará. Two 10-day expeditions were conducted in May and September 2015. Sampling was conducted in three distinct areas of the forest fragment: the interior, edge, and matrix. The matrix comprised a (peridomiciliary area with domestic animal breeding facilities (chicken, pig, and cattle sheds) and plantations extending to the forest edge). The distance between the forest edge and the interior was 600 m.

Three collection techniques were employed: flannel dragging, “walking trap” or flannel leggings, and an attractive CO_2_ trap. Collections were conducted consistently during the early morning hours (6:30-9:30 am). In the dragging with flannel technique, a white flannel (1.50 m × 0.80 m) attached to a wooden stick (0.85 m) and tied to a string at the front end was dragged over the vegetation along the trails. The “walking trap” technique involved walking through the vegetation while wearing white flannel leggings to capture questing ticks that might be on the vegetation awaiting a host. Every five steps along the trails, the flannel used for dragging was inspected, and any ticks present were individually placed in tubes containing isopropyl alcohol, except for larval clusters, which were removed using adhesive tape (before the larvae were dispersed across the flannel) and placed in tubes containing isopropyl alcohol. The leggings were also inspected for the presence of ticks. For the CO_2_ trap, 300 mL of 20% lactic acid and 125 g calcium carbonate were used. The CO_2_ traps were set up on alternate days for two hours in the morning at two fixed points 300 m apart in each area. A sampling effort of 60 leggings per expedition (six leggings per day × 10 days total) and 30 drags per expedition (three drags per day × 10 days total) was achieved. For the attractive CO_2_ traps, the sampling effort was 30 traps per expedition (six traps per day for five days).

The larvae, nymphs, and adults were counted individually. Adults and nymphs were morphologically identified following the method described by Dantas-Torres et al. (2019). Individual larvae were identified through molecular analysis, whereas larvae collected in clusters were treated as a single unit, with a pool of five larvae separated for molecular identification, following Ogrzewalska et al. (2009). DNA was extracted from tick larvae using the boiling technique, whereas nymph and adult DNA were extracted individually using the phenol/phenol-chloroform method. Conventional polymerase chain reaction (PCR) was performed for molecular identification of tick larvae at the species level and to investigate microorganisms of the genera *Rickettsia*, *Ehrlichia*, *Anaplasma,* and *Borrelia* in all extracted tick DNA samples (individual ticks and pools). Specific primers were used for each agent and the annealing temperature (AT) of each primer was optimized. Table 1 presents the primers used, target genes, PCR techniques, amplified product sizes, and the reference protocols used. Each reaction included DNA from *Rhipicephalus microplus* (BME2 cell line culture), *Rickettsia parkeri* strain at24 (culture), *Borrelia anserina* strain AL (culture), *Babesia bigemina* (bovine positive), *Anaplasma platys* (dog positive), and *Ehrlichia canis* (dog positive), along with two negative controls (ultrapure water).

**Table 1:** Sequences of the primers used, along with the respective organisms, target genes and size of the amplified fragment.

**Organisms/Gene/*primers***	**Molecular assay**	**Aim**	**Sequence (5’-3’)**	**Fragment**	**AT**	**Reference**
Ixodidae Family						
**16SrRNA**	cPCR	Characterization				
16S+			CCGGTCTGAACTCAGATCAAGT	460 pb	55 °C	Mangold et al. (1998)
16S-			GCTCAATGATTTTTTAAATTGCTGT			
*Rickettsia* spp.						
***glt*A**	cPCR	Characterization				
CS239 F			GCTCTTCTCATCCTATGGCTATTAT	834 pb	52 °C	Labruna et al. (2004)
CS1069 R			CAGGGTCTTCGTGCATTTCTT			
*Ehrlichia* spp.						
***dsb***	snPCR	Screening				
DSB-330			GATGATGCTTGAAGATATSAAACAAAT	349 pb	52 °C	Almeida et al. (2013)
DSB-380			ATTTTTAGRGATTTTCCAATACTTGG			
DSB-720			CTATTTTACTTCTTAAAGTTGATAWATC			
*Borrelia* spp.						
***fla*B**	nPCR	Characterization				
*BorFlaF1*			TACATCAGCTATTAATGCTTCAAGAA	740 pb	55 °C	Blanco et al. (2017)
*BorFlaR1*			GCAATCATWGCCATTGCRGATTG			
*BorFlaF2*			CTGATGATGCTGCTGGWATGG		55 °C	
*BorFlaR2*			TCATCTGTCATTRTWGCATCTT			
*Anaplasma* spp*.*						
**16S rRNA**	nPCR	Characterization				
*ge3A*			CACATGCAAGTCGAACGGAT TATTC	546 pb	55 °C	Massung et al. (1998)
*ge10R*			TTCCGTTAAGAAGGATCTAATCTCC			
*ge9F*			AACGGATTATTCTTTATAGCTTGCT			
*ge2*			GGCAGTATTAAAAGCAGCTCCAGG			

cPCR: conventional PCR; nPC: nested PCR; snPC: semi-nested PCR; AT: annealing temperature.

All PCR were performed in a model T100 thermocycler (Bio-Rad®), following the protocols specified for each gene (Table 1). The PCR products were analyzed via gel electrophoresis (1.5% agarose), stained with ethidium bromide, and visualized using a UV Transilluminator. PCR-positive samples were purified using ExoSAP® and sequenced using the SANGER method. The sequences obtained were compared to those deposited in GenBank.

## Results

A total of 561 ticks were captured, comprising 525 larvae, 29 nymphs, and seven adults. The majority (76.6%; 430/561) originated from the interior of the forest fragment (Table 2). Eight species were identified, all of which were classified under the genus *Amblyomma* (Table 2). Molecular identification of larvae from the three clusters and 27 individual larvae was unsuccessful because of insufficient DNA or the formation of non-specific bands, which remained at the genus level (Table 2).

**Table 2:** Distribution of free-living tick species collected in the forest fragment from municipality of Peixe-Boi-PA, according to different areas (Inside, Edge and Matrix).

**Tick species**	**Forest Fragment**			**Total**
	**Inside**	**Edge**	**Matrix**	
*A. cajennense* s.s.	1 L	2 M, 1 F, 1 L	1 M, 3 N	9
*A. naponense*	2 M, 1 L, 6 N, 45 CL	11 N, 1 L	-	66
*A. humerale*	4 N, 4 L	6 L	-	14
*A. calcaratum*	2 N, 3 L, 103 CL	1 N	-	109
*A. pacae*	1 M	-	-	1
*A. varium*	1N, 3 L	2 N, 1 L, 63 CL	3 L	73
*A. goeldii*	1 L, 36 CL	1 L	-	38
*A. geayi*	1L	-	-	1
*Amblyomma* spp.	85 CL, 116 CL, 15 L	11 L	22 CL, 1 L	250
**Total**	430	101	30	561

M: male; F: female; N: nymph; L: larva; CL: Larvae collected within one cluster.

Regarding the distribution of tick species within the forest, eight species were identified, with six collected at the edge and only two in the matrix. Unidentified *Amblyomma* species were collected from all the three samples (Table 2).

Molecular analysis targeting amplification of the *glt*A gene, present in *Rickettsia* spp., revealed that the two samples contained rickettsial DNA. These corresponded to an *Amblyomma varium* larva captured in an urban-rural area and an *Amblyomma goeldii* larva captured from the forest edge. The fragments were sequenced and analyzed for similarity to sequences from other species deposited in GenBank. Sequencing of the amplicons generated for both *A. varium* and *A. goeldii* demonstrated 100% similarity to *Rickettsia bellii* (DQ865204 and MK9626971, respectively). The GenBank nucleotide sequence accession numbers for the partial sequences generated in this study are MT012706 and MT012707. All tested samples were negative for *Ehrlichia*, *Borrelia,* and *Anaplasma*.

## Discussion

In the present study, *R. bellii* DNA was identified in two tick species. This bacterium has recently been detected in ticks from various regions of Latin America (Labruna et al., 2011). Given that this rickettsial agent is frequently found in *Amblyomma* spp., some researchers have proposed the existence of symbiotic co-evolution between ticks and rickettsiae (Labruna et al., 2004). Although the pathogenicity of this rickettsial agent remains unknown (Labruna et al., 2011), La Scola et al. (2009) observed the formation of eschars in guinea pigs after experimental infection.

In Brazil, *R. bellii* has been detected in several *Amblyomma* species, including *Amblyomma auricularium*, *Amblyomma aureolatum*, *Amblyomma cajennense s.s.*, *Amblyomma dubitatum, Amblyomma humerale*, *Amblyomma incisum, Amblyomma longirostre*, *Amblyomma naponense*, *Amblyomma nodosum*, *Amblyomma oblongoguttatum*, *Amblyomma ovale, Amblyomma rotundatum* and *Amblyomma scalpturatum* (Labruna et al., 2004; Brites-Neto et al., 2015). This is the first report of *R. bellii* in *A. varium* and *A. goeldii* ticks in Brazil. To date, there has been only one record of *R. bellii* in *A. varium* documented by Ogrzewalska et al. (2012) in Peru, where the larvae of this tick species were collected from birds. The tick *A. goeldii* remains understudied because of the scarcity of specimens found on its hosts, particularly in immature stages (Martins et al., 2015), resulting in limited knowledge about the hemoparasites harbored by this species.

*Ehrlichia*, *Anaplasma,* and *Borrelia* were not detected in the ticks examined in this study. Similarly, some studies have failed to detect *Ehrlichia* in ticks (Gruhn et al., 2019), suggesting a lack of evidence for infection by this pathogen in *Amblyomma* in Brazil (Gruhn et al., 2019). However, there have been reports that *Anaplasma* spp. and *Ehrlichia* spp. ticks of the genus *Amblyomma* parasitize wild animals (Sousa et al., 2018).

It should be considered that this positivity may be related to the remnants of the blood meal of the vector arthropod in the infected host (Sousa et al., 2018). Similarly, Ogrzewalska et al. (2019) did not detect piroplasmids in clustered ticks but emphasized the importance of further research and did not rule out the possible circulation of these pathogens among tick species in Brazil. It is also important to continue investigating *Borrelia* spp. in ticks, as *Borrelia* sp. DNA has been detected in *Amblyomma* ticks in Brazil (Pacheco et al., 2019) and Argentina (Cicuttin et al., 2019). Additionally, Araújo et al. (2022) detected *Borrelia* spp., genetically similar to *Borrelia miyamotoi* and *Borrelia lonestari,* in *Amblyomma calcaratum*, a species identified in our study. In conclusion, this study marks the first record of *R. bellii* in *A. varium* and *A. goeldii* ticks in Brazil.
